# Spinal osteoarthritis is a risk of vertebral fractures in postmenopausal women

**DOI:** 10.1038/s41598-024-53994-1

**Published:** 2024-02-12

**Authors:** Tatsuhiko Kuroda, Masataka Shiraki, Mitsuru Saito, Tomohiko Urano

**Affiliations:** 1 Public Health Research Foundation, Shinjuku-ku, Tokyo Japan; 2 Research Institute and Practice for Involutional Diseases, Azumino City, Nagano Japan; 3 Department of Orthopedic Surgery, Tokyo Jikei University, School of Medicine, Minato-ku, Tokyo Japan; 4 Department of Geriatric Medicine, International University of Health and Welfare School of Medicine, 4-3, Kozunomori, Narita City, Chiba 286-8686 Japan

**Keywords:** Osteoarthritis, Fracture risk, Bone mineral density, Epidemiology, Osteopetrosis

## Abstract

Recent studies have revealed that despite high bone mineral density (BMD), osteoarthritis (OA) is a risk factor for osteoporotic fractures. However, the relationship between spinal OA and vertebral fractures has not yet been fully investigated. This longitudinal analysis used a subset of ongoing cohort study consist with Japanese postmenopausal women. The prevalence of spinal OA was determined using Kellgren–Lawrence grading method. The incidence of vertebral fractures were determined by semiquantitative analysis of spinal X-ray films. The relationship between the presence of spinal OA and incidence of vertebral fractures was evaluated using the Cox regression analysis. In total, 1480 women were followed up for 8.1 ± 6.4 years. Among them, 923 were diagnosed with spinal OA, and incident vertebral fractures were observed in 473 participants. After adjusting for confounding variables, the spinal OA (≥ grade 2) was a significant predictor of incident vertebral fractures (hazard ratio, 1.52; 95% confidence interval: 1.19–1.93, *p* = 0.001). Using ROC analysis, the thresholds of lumbar BMD for incident vertebral fractures were 0.952 g/cm^2^ for patients with spinal OA and 0.753 g/cm^2^ for patients without spinal OA. The presence of spinal OA is a risk factor for incident vertebral fractures despite high lumbar BMD.

## Introduction

Osteoporosis is a systemic and metabolic skeletal disease characterized by decreased bone mineral density (BMD), compromised bone strength, and an increased risk of fractures. With an aging population worldwide, preventive measures against osteoporosis and fragility fractures have become increasingly important. Since post fracture deformities are attributed to the clinical manifestations of osteoporosis, the primary endpoint of osteoporosis treatment is the prevention of osteoporotic fractures. Therefore, identifying various types of fracture risks to predict fracture susceptibility is critical. In our cohort study, we identified several risk factors of osteoporotic fracture^1–5^.

Osteoarthritis (OA) is a chronic degenerative joint disease that involves the breakdown of articular cartilage in almost any joint of the body and most frequently affects middle-aged or older people. Osteoarthritic deformity is observed not only in the cartilage, but also in the periarticular bone surface or trabecular bone area near the joint. The main symptoms of OA are stiffness, pain, and impaired joint movement, all of which deteriorate health-related quality of life in older adults^6,7^. Among the various types of OA, knee, hand, and hip joint OA have been the subject of many clinical investigations. However, there has been far less achievement in spinal OA^8^. The local manifestations of spinal OA on X-ray films indicate disc space narrowing, osteophytosis, endplate sclerosis, or facet joint deformity. These abnormalities in the vertebral column lead to deterioration of vertebral alignment and an increase in pressure on the spinal cord and/or nerve roots. This process of OA in the vertebral column induces pain in the lower back and numbness of the legs, which deteriorate posture and the ability to locomote in patients with severe spinal OA^9^. Treatments for OA to reduce pain and improve joint movement include exercise, physical therapies, nutritional intervention, and analgesics^10–12^. However, these treatments are less effective and cannot cure spinal OA, which makes the clarification of the pathophysiology of spinal OA a critical issue.

Since the bone manifestations of OA are accompanied by high BMD^13,14^, it is uncertain or rather negative whether OA is associated with osteoporotic fracture^15–20^. The Women’s Health Initiative study group was the first to report that the age-adjusted hazard ratio of osteoporotic fractures was significantly higher in patients with OA than in those without OA^21^. In addition, a large, multinational cohort study (the GLOW study; Longitudinal Study of Osteoporosis in Women) was conducted to clarify the effect of comorbidities on fracture risk. OA was identified as a significant risk for incident fracture (hazard ratio [HR]: 1.3, 95% confidence interval [CI]: 1.2–1.4, *p* < 0.001) after adjustment for age^22^. More recently, a huge retrospective cohort study was conducted in the United Kingdom including approximately 260,000 patients for 10 years of observation, and the results indicated a significantly higher susceptibility of fractures in patients with OA^23^. These reports suggest that OA is a potential risk factor for osteoporotic fractures. However, these studies mainly focused on the relationship between self-reported OA and long bone fractures, and the site of OA was either not identified or included multiple sites, such as the knee, hip, or wrist joints. However, there are no reports on the relationship between spinal OA and vertebral osteoporotic fractures. Moreover, there is no compelling evidence related to the mechanisms why OA patients have a higher susceptibility to fractures, despite their higher BMD.

Therefore, we investigated whether spinal OA is an independent risk factor for osteoporotic fractures in Japanese postmenopausal women and which bone parameters are independently associated with the incidence of vertebral fractures.

## Methods

### Participants

The participants in this study were a subset of those enrolled in the Nagano Cohort Study, an ongoing registration study of postmenopausal female out-patients who had visited a primary care institute in Nagano Prefecture, Japan, since 1993. The Nagano cohort study included the clinical records of ambulatory women who received treatment at a primary care institution^1–5^.

The flowchart of the study population and the selection for this analysis were shown in Supplementary Fig. 1. Patients were asked to confirm their willingness to participate in the Nagano cohort, and baseline survey was conducted on subjects who have consented, and thereafter, information such as BMD and fractures were periodically followed up. To date, this cohort has enrolled more than 2200 subjects.

For this analysis, postmenopausal women who were aged ≥ 50 years were selected. Women with acute or serious infectious illnesses (such as pneumonia), terminal cancer, or secondary osteoporosis (such as long-term steroid use, primary hyperparathyroidism, or postpartum osteoporosis) at baseline were excluded from the present analysis. We also excluded the patients who started glucocorticoids after the baseline assessment. In the current investigation, our initial cohort comprised 67 individuals (constituting 2.9% of the 2284 registered participants) who reported smoking, and 220 individuals (9.6% of the total 2284 registered participants) who identified as drinkers. These proportions indicate potential limitations in statistical power for the assessment of these lifestyle factors on fractures. Patients with terminal stage chronic renal failure (eGFR < 20 ml/min/1.73m^2^) were also excluded. Subjects who did not adhere to the follow-up protocol were excluded from the study. Inclusion criteria comprised subjects who were followed for more than one year. Additionally, participants who experienced the first incident fracture within one year from the initial registration were also included in the analysis. Standard treatment for the participants’ comorbidities, including osteoporosis, was provided during the observation. The follow-up period ended with the occurrence of the first incident of osteoporotic fracture, death of participants, or if it was impossible to follow up due to relocation, institutionalization, or referral to another hospital, whichever occurred first.

The study protocol was reviewed by the ethics committee of the Research Institute and Practice for Involutional Diseases, Japan prior to commencement, and the study was conducted in accordance with the principles of the Declaration of Helsinki. Comprehensive written informed consent was obtained from all participants.

### Assessment of bone parameters

BMD at the L2 to L4 lumbar spine (L-BMD) and total hip (H-BMD) were measured using dual-energy X-ray absorptiometry (GE Healthcare, WI, USA), and a quality assurance test was carried out on every measurement to detect machine drift. The interassay variance of the L-BMD measurement in our laboratory was 0.5 ± 0.5% (CV ± SD). Serum 25-hydroxyvitamin D (25 [OH] D) levels were measured using ECLIA. The serum level of calcium was corrected (cCa) based on the serum albumin level using the following formula: cCa = (Calcium + 4)-albumin. Serum levels of bone-derived alkaline phosphatase (BAP; Ostase CLEA; Beckman Coulter, Atlanta, GA, USA) and urinary excretion of type I collagen-cross-linked N-telopeptides (NTx; Osteomark; Creative Diagnostics, NY, USA) were also measured. The urinary excretion of pentosidine and plasma levels of total homocysteine were measured using an HPLC system after hydrolysis of the urine samples^3,4^. Plasma levels of total homocysteine were measured using an HPLC system. All chemical parameters mentioned above were measured at the central laboratory (LSI Medience, Tokyo, Japan).

### Radiographic assessment of vertebral fracture

Prevalent fractures were defined as fractures occurring in the vertebrae, ribs, pelvis, proximal end of the humerus, distal end of the radius, clavicle, proximal end of the femur, proximal or distal end of the tibia, or fibula at baseline. The participants were asked about their history of long bone fractures during the interview or their medical records. Vertebral fractures were identified through a semiquantitative analysis of radiographs of the thoracic and lumbar spine at both baseline and follow-up, following a 24-month interval^24^. When the subject complained back pain or falling, additional X-ray evaluation was carried out on demand. The X-ray films were evaluated for the presence and severity of vertebral fractures using Genant’s semiquantitative (SQ) method^24^. The SQ grade 1 (mild deformity) or more (Grade 2 and 3, moderately and severely deformed, respectively) were judged as fractured vertebrae. The judgment of vertebral fractures in the present study was independently assessed by at least two experienced physicians and when the judgment was disparate, the adjudication was made among the members^25^.

### Assessment of spinal osteoarthritis

Spinal OA was assessed through radiographic examination of the T4–L4 vertebral column. The degree of degeneration was evaluated using the Kellgren–Lawrence (KL) grading system^26^. To combine multiple joint assessments into a single KL grade, we judged patients’ KL grade based on both the degree and extent of pathological changes. To validate our scoring system, the OA grading scores in the present study were compared with the scores obtained from another system^27–29^. Subjects were classified into two groups: without spinal OA (KL grade 0 and 1) and with spinal OA (≥ grade 2). Given our specific focus on the relationship between vertebral body fracture and vertebral body OA, emphasize features such as endplate sclerosis, narrowing disc space, and osteophytosis rather than facet joints. To consolidate assessments across multiple joints (T4-L4) into a unified KL grading, we considered both the degree and extent of pathological changes^27^.

In our postmenopausal women cohort, nearly all subjects exhibited possible osteophyte lipping, resulting in sporadic occurrences of KL grade 0 (G0) cases. Given the subtle pathological changes in degeneration and deformation observed in G1, we determined that there were no substantive differences between G0 and G1. Consequently, we combined all G0 and G1 cases, defining them as G1 (not/doubtful spinal OA cases), with G2 or higher designated as spinal OA. Detailed information on our OA assessment has been previously presented^28,29^. When our K-L grading was compared with the alternative evaluation system proposed by Oishi et al.^27^, the number of joints with endplate sclerosis in Grades 1 to 4 were as follows (means + SD): 0.1 ± 0.3 (Grade 1), 0.1 ± 0.4 (Grade 2), 0.5 ± 0.8 (Grade 3), and 1.4 ± 1.7 (Grade 4). For joints exhibiting osteophytosis, the values were 2.8 ± 3.0 (Grade 1), 5.6 ± 4.0 (Grade 2), 6.4 ± 3.3 (Grade 3), and 7.8 ± 3.3 (Grade 4). In cases of disc space narrowing, the figures were 1.1 ± 1.1 (Grade 1), 1.6 ± 1.6 (Grade 2), 2.4 ± 1.8 (Grade 3), and 4.9 ± 2.8 (Grade 4). Following the evaluation of these three pathological findings, the highest grade was selected as the representative score for the subject's osteoarthrosis^29^. The judgment of OA grading was independently assessed by at least two experienced physicians and when the judgment was disparate, the adjudication was made among the members.

### Treatment of comorbidities and osteoporosis

All comorbidities present at baseline and during the observation period were properly treated, with informed consent from the participants. Osteoporosis has also been treated with antiresorptive drugs, such as estrogen, SERM, bisphosphonates, or denosumab. Teriparatide treatment lasting for a short period (1–2 years) was also considered as a treatment term. If the treatment period exceeded half of the observation period, we defined the patient as being under treatment for osteoporosis. Native vitamin D or hormonal forms of vitamin D analogs, such as 1α(OH)D_3_, were not considered as treatment of osteoporosis because of the lack of robust evidence for fracture prevention.

### Statistical analysis

Numerical data were presented as the mean ± SD, while categorical data were expressed as numbers and proportions (%). The baseline age, body mass index, BMD, and bone parameters were compared using analysis of variance or χ^2^ test between participants with or without incident vertebral fracture. In the longitudinal analysis, the Cox proportional hazards model was used to estimate the HRs and 95% CIs of vertebral fracture incidence, with adjustments for candidate risk factors. Candidate factors were selected from those that were significantly different (*p* < 0.05) between patients with or without incident fractures and were finally extracted using the stepwise method. The Kaplan–Meier curves were plotted to illustrate the survival curve of the incidence of fractures during the observation period, and a log-rank test was used to assess the statistical significance of the differences between patients with and without baseline spinal OA. The relationship between KL grade and the time of incident fracture was evaluated by median survival time. To determine the fracture thresholds of baseline L-BMD in patients with or without spinal OA, a receiver operating characteristic (ROC) analysis was conducted. All comparisons were two-sided, and *p* values < 0.05 were considered statistically significant. Data were analyzed using JMP version 16.0 (SAS Institute, Cary, NC, USA).

## Results

In total, 1480 participants were selected from the cohort for analysis. Among of them, 473 participants (32.0%) were diagnosed with osteoporotic incident vertebral fractures.

Table 1 summarizes the baseline characteristics of the participants with or without incident vertebral fractures. Age, L-BMD, H-BMD, NTx, BAP, pentosidine, and homocysteine levels were significantly different between participants with and without incident vertebral fractures (*p* < 0.05). The proportions of osteoporosis treatment, prevalent vertebral fractures, and spinal OA at baseline were also significantly different between participants with and without incident vertebral fractures (*p* < 0.05). The observation period for participants with incident fractures was significantly shorter (6.8 ± 5.4 years) than that for those without fractures (9.3 ± 7.0 years, *p* < 0.001).

**Table 1:** Baseline characteristics of the participants with or without incident vertebral fracture.

Item	Total (n = 1480)	Incident vertebral fracture		*p*
		No (n = 1007)	Yes (n = 473)	
Age, years old	65.5 $\pm$ 9.3	64.2 $\pm 9.2$	68.4 $\pm 8.7$	< 0.001
BMI, kg/m^2^	22.6 $\pm$ 3.2	22.6 $\pm 3.2$	22.7 $\pm 3.2$	0.463
L-BMD, g/cm^2^	0.924 $\pm$ 0.195	0.968 $\pm 0.192$	0.854 $\pm 0.182$	< 0.001
H-BMD, g/cm^2^	0.755 $\pm$ 0.135	0.779 $\pm 0.132$	0.708 $\pm 0.130$	< 0.001
25(OH)D, ng/mL	20.7 $\pm$ 6.0	20.9 $\pm 6.1$	20.4 $\pm 5.9$	0.189
cCa, mg/dL	9.1 $\pm$ 0.5	9.1 $\pm 0.5$	9.1 $\pm 0.5$	0.969
Phosphate, mg/dL	3.5 $\pm 0.5$	3.5 $\pm 0.5$	3.4 $\pm 0.5$	0.093
NTx, nM/mMCr	53.6 $\pm$ 29.3	51.4 $\pm 25.6$	58.5 $\pm 35.5$	< 0.001
BAP, IU	31.9 $\pm$ 12.5	31.2 $\pm 11.8$	33.2 $\pm 13.7$	0.009
Pentosidine, pM/mgCr	47.4 $\pm$ 26.1	44.3 $\pm 23.5$	54.0 $\pm 29.8$	< 0.001
Homocysteine, nM/mL	9.4 $\pm$ 3.5	9.1 $\pm 3.3$	10.0 $\pm 3.9$	< 0.001
Treatment of osteoporosis, yes (%)	546 (36.9)	349 (34.6)	197 (41.6)	0.028
Prevalent vertebral fracture, yes (%)	289 (19.5)	118 (11.7)	171 (36.2)	< 0.001
Spinal OA, yes (%)	923 (62.3)	571 (56.7)	352 (74.4)	< 0.001
Observation period, years	8.1 $\pm$ 6.7	9.3 ± 7.0	6.8 ± 5.4	< 0.001

BMI, Body mass index; L-BMD, Lumbar bone mineral density; H-BMD, Total hip bone mineral density; cCa, Corrected calcium; NTX, Type I collagen cross-linked N-telopeptide; BAP, Bone-derived alkaline phosphatase; OA, Osteoarthritis.

The Cox proportional hazards model was used to determine the independent risk factors for incident vertebral fractures. As shown in Table 2, after adjusting for confounding variables, age, L-BMD, pentosidine use, and the prevalence of vertebral fracture significantly contributed to the incidence of vertebral fracture. The presence of spinal OA (≥ grade 2) was a significant independent predictor of incident vertebral fracture occurrence (HR: 1.52; 95% CI 1.19–1.93, *p* = 0.001).

**Table 2:** Cox hazard model for the incident vertebral fracture.

Item	HR	95% CI		*p*
Age, 10 years up	1.51	1.31	1.73	< 0.001
L-BMD, 0.1 g/cm^2^ up	0.79	0.74	0.85	< 0.001
NTX, 10 nM/mMCr up	1.02	0.99	1.05	0.213
Pentosidine, 10 pM/mgCr up	1.04	1.01	1.06	0.026
Homocysteine, 1 nM/mL up	1.01	0.98	1.04	0.481
Treatment of osteoporosis, yes	0.85	0.68	1.07	0.165
Prevalent fracture, yes	2.32	1.83	2.95	< 0.001
Spinal OA, yes	1.52	1.19	1.93	0.001

HR, Hazard ratio; 95% CI, 95% Confidence interval; L-BMD, Lumbar bone mineral density; NTX, Type I collagen cross-linked N-telopeptides; OA, Osteoarthritis.

**Figure 1:**
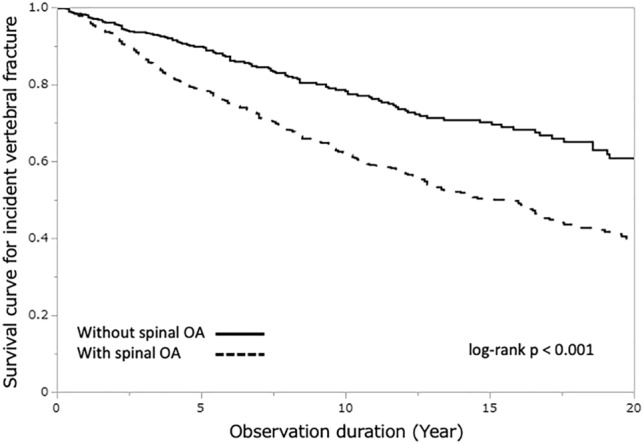
Kaplan–Meier plot for incident vertebral fracture by the participants with or without spinal osteoarthritis.

The Kaplan–Meier plot of the incidence of vertebral fractures during the observation period is shown in Fig. 1. The log-rank test showed that participants with spinal OA (≥ grade2) indicated a significantly higher and faster occurrence of incident vertebral fractures than participants without spinal OA (*p* < 0.001). The median survival time by KL grade was grade 1 = 26.8 years, grade 2 = 16.2 years, grade 3 = 13.0 years, and grade 4 = 12.6 years.

**Figure 2:**
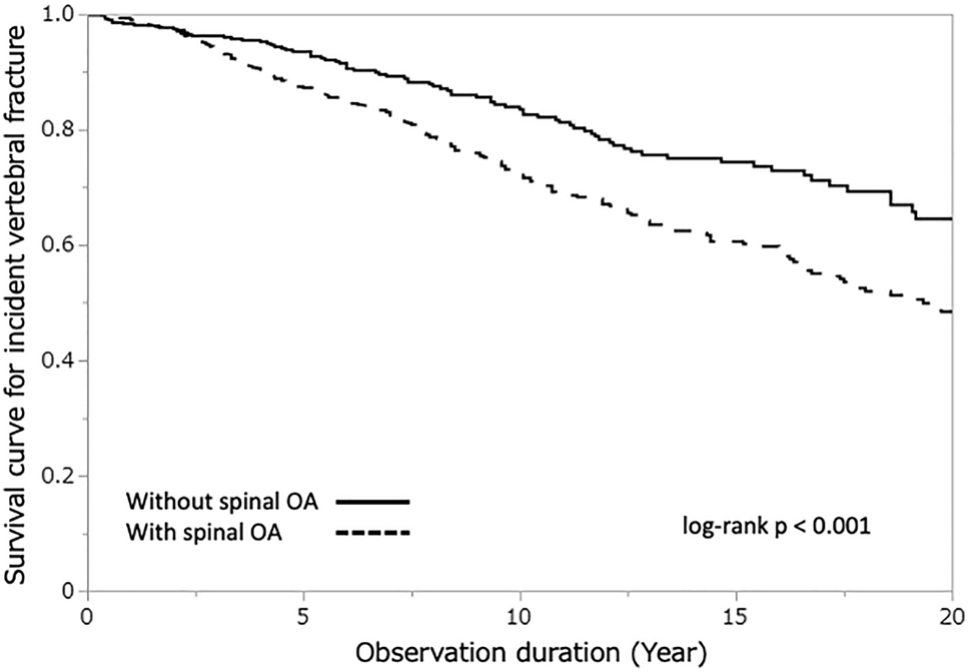
Kaplan–Meier plot for incident vertebral fracture by the participants with or without spinal osteoarthritis (without prevalent vertebral fracture, n = 1191).

**Figure 3:**
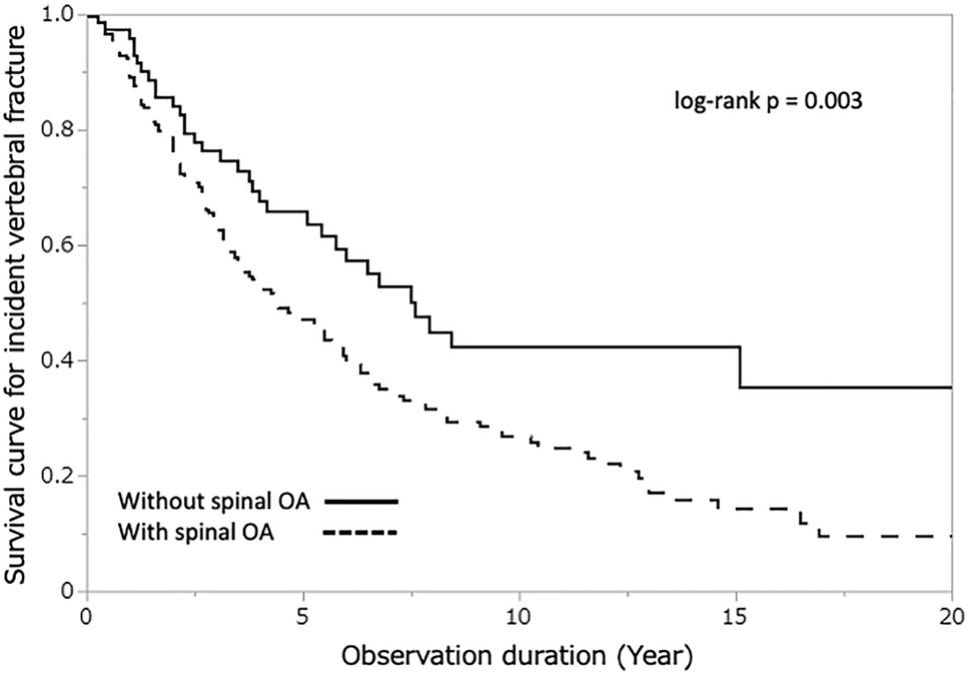
Kaplan–Meier plot for incident vertebral fracture by the participants with or without spinal osteoarthritis (with prevalent vertebral fracture, n = 289).

The most powerful predictor of incident vertebral fracture was the presence of prevalent vertebral fractures (HR, 2.32; 95% CI 1.83–2.95, *p* < 0.001). Prevalent fractures are known to be associated with secondary OA, commonly referred to as post-fracture OA^29^. To investigate whether both secondary and primary OA are recognized as risk factors for incident fractures, the Kaplan–Meier plots for incident vertebral fracture divided by the prevalence of vertebral fracture are presented. Participants with spinal OA had significantly higher incident fracture rates than those with prevalent vertebral fractures (Fig. 2, *p* < 0.001) or without prevalent vertebral fractures (Fig. 3, *p* = 0.004). These findings suggest that both secondary and primary OA are significant risk factors for vertebral fractures.

ROC analysis was performed to clarify the baseline thresholds of L-BMD for incident vertebral fractures in participants with and without spinal OA. The thresholds for incident vertebral fractures in participants with and without spinal OA were 0.952 and 0.753 g/cm^2^, respectively. The corresponding T scores for these L-BMD values were − 1.7 SD and − 3.1 SD, respectively. Thus, the fracture threshold in the participants with spinal OA was 26.4% higher than that in participants without spinal OA.

## Discussion

Osteoporosis and OA are common skeletal disorders in older individuals. Several decades ago, these two disorders were recognized as distinctly different because BMD, a major determinant of bone strength, is the opposite between osteoporosis and OA^16,30,31^. In individuals with OA, BMD was found to be high not only in the neighboring joint, but also in joints distant from the measured BMD sites, suggesting that metabolic or genetic processes may exist to achieve high BMD in OA^29,32^. However, several recent reports have found that OA is an independent risk factor for osteoporotic fracture^21–23^. Additionally, thoracolumbar fractures caused by osteoporotic fractures are often accompanied by kyphosis with disc or ligament damage^30^, which can lead to vertebral OA. These observations suggested a possible transition between OA and osteoporotic fractures. Recent reports have shown that OA at the knee or hip joints is associated with fractures of the femur or vertebrae^16–18^. These findings suggest a possible deterioration of bone strength at the site of the bone affected by OA, even though BMD may be higher in those sites than in regions without OA.

In this study, we aimed to investigate whether the presence of OA in the bone leads to the deterioration of bone strength, particularly in the spine, as no previous studies have explored the relationship between spinal OA and osteoporotic vertebral fractures. Our study findings indicated that the presence of spinal OA was independently associated with incident vertebral fractures (HR: 1.52; 95% CI 1.19–1.93, *p* = 0.001). A significant association between spinal OA and incident vertebral fractures was observed in participants with and without prevalent vertebral fractures.

The diagnosis of osteoporosis has traditionally relied on the measurement of BMD, with a threshold of − 2.5 SD in T-score defining osteoporosis and a BMD range of − 1.0 to − 2.49 SD in T-score defining osteopenia, as proposed by the World Health Organization (WHO)^33,34^. However, osteoarthritic changes in the vertebrae are present in approximately 75% of older men and 60% of women, whereas OA at the hip joint is present in around 30% of both men and women^34^. WHO has recommended the use of hip BMD in diagnostic criteria for osteoporosis due to the potential overestimation of BMD in existing OA^34^. In the present study, we estimated the threshold of L-BMD to incident vertebral fracture using ROC analysis, and the thresholds of—L-BMD values in participants with or without OA were markedly different, with values of 0.952 g/cm^2^ (− 1.7 SD) and 0.643 g/cm^2^ (− 3.1 SD), respectively. A higher fracture threshold in patients with spinal OA may indicate that BMD does not correlate with bone strength. In contrast, the fracture threshold in participants without spinal OA was far lower than the BMD diagnostic criteria for osteoporosis, likely because most patients with osteoporosis in the present study were mainly treated with bisphosphonates, SERMs, or denosumab, which can lower the fracture threshold.

In contrast, the levels of pentosidine were a significant risk for incident vertebral fracture (HR: 1.04, 95% CI 1.01–1.06, *p* = 0.026, Table 2). Pentosidine is a well-known risk factor for osteoporotic fractures^3,4^ and is known to cause bone fragility independent of BMD. Therefore, pentosidine may be a risk factor for incident fractures even in spinal OA with high BMD. In addition, a higher level of homocysteine has also been reported as a risk factor for osteoporotic fractures^36,37^. Our previous research indicated that serum homocysteine concentration is an independent risk factor for the progression of vertebral OA in postmenopausal women^29^. Therefore, higher levels of homocysteine observed at baseline in participants with an incident vertebral fracture may be a risk factor for the progression of osteoarthritis.

This study had several limitations that need to be acknowledged. First, the study population was relatively small and included only Japanese women; therefore, caution should be exercised when generalizing the present results to a wider population, including both sexes and various ethnicities. Second, selection bias might have occurred because the investigation was conducted in a subset of the Nagano cohort population in Japan. However, the baseline data of the current study were almost identical to those of our previous studies in terms of participant age, body composition, and BMD. Furthermore, these basic data were comparable to those of the general Japanese population, as determined by propensity-score analysis^37^, suggesting that the selection bias was likely minimal. Third, some patients were undergoing treatment for osteoporosis, which could have potentially underestimated the effect of OA on fracture incidence. Fourth, in the present study, we disregarded facet joint OA in the analysis, and this might result in underestimating the impact of OA on BMD. Finally, in this paper, we analyzed the association between fracture and osteoarthritis in the thoracic and lumbar spine together. Furthermore, it is desirable to increase the number of cases and analyze the association separating thoracic and lumbar spine, respectively.

## Conclusion

Our study demonstrated that spinal OA was a significant independent predictor of vertebral osteoporotic fractures after adjusting for confounders, such as age, L-BMD, prevalent vertebral fractures, and pentosidine. However, the higher fracture threshold of L-BMD in participants with spinal OA indicates that treatment decisions should be carefully determined.

### Supplementary Information

Supplementary Figure S1.

Supplementary Legends.

## Data Availability

This datasets used and analyzed during the current study available from the corresponding author on reasonable request.
