# Supplementary figures and images for: Spinal osteoarthritis is a risk of vertebral fractures in postmenopausal women
Source: Sci Rep. 2024 Feb 12;14:3528. doi: 10.1038/s41598-024-53994-1 (PMC10861596; doi:10.1038/s41598-024-53994-1)

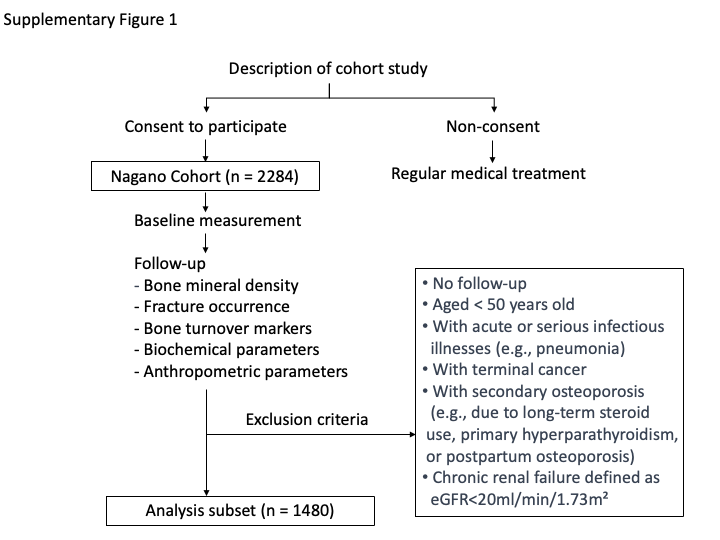

Supplement: Supplementary file 1 — Supplementary Figure S1. [file 41598_2024_53994_MOESM1_ESM.tiff]
