# Supplementary material for: Spinal osteoarthritis is a risk of vertebral fractures in postmenopausal women
Source: Sci Rep. 2024 Feb 12;14:3528. doi: 10.1038/s41598-024-53994-1 (PMC10861596; doi:10.1038/s41598-024-53994-1)
Supplement: Supplementary file 2 — Supplementary Legends. [file 41598_2024_53994_MOESM2_ESM.docx]

**Supplementary Figure Legends**

Supplementary Figure 1. The flowchart of the study and the selection for this analysis.
